# High *Mycoplasma pneumoniae* loads and persistent long-term *Mycoplasma pneumoniae* DNA in lower airway associated with severity of pediatric *Mycoplasma pneumoniae* pneumonia

**DOI:** 10.1186/s12879-019-4667-y

**Published:** 2019-12-10

**Authors:** Jinrong Liu, Fei Zhao, Jie Lu, Hui Xu, Hui Liu, Xiaolei Tang, Haiming Yang, Jianzhong Zhang, Shunying Zhao

**Affiliations:** 1 Department of Respiratory Medicine II, Beijing Children’s Hospital, Capital Medical University, National Center for Children’s Health, No. 56, Nanlishi Road, Xicheng District, Beijing, 100045 China; 2 National Institute for Communicable Disease Control and Prevention, Chinese Center for Disease Control and Prevention, State Key Laboratory of Infectious Disease Prevention and Control, Beijing, 102206 China; 3 Key Laboratory of Major Diseases in Children, Beijing Key Laboratory for Pediatric Diseases of Otolaryngology, Head and Neck Surgery, Beijing Pediatric Research Institute, Beijing Children’s Hospital, Capital Medical University, National Center for Children’s Health, Beijing, 100045 China; 4 Biobank for Clinical Data and Samples in Pediatric, Beijing Pediatric Research Institute, Beijing Children’s Hospital, Capital Medical University, National Center for Children’s Health, Beijing, 100045 China

**Keywords:** Refractory, Mycoplasma pneumoniae, Lower airway, Loads, Duration

## Abstract

**Background:**

An increased number of refractory *mycoplasma pneumoniae* (MP) pneumonia (MPP) cases have been reported. However the duration of MP infection in lower airway and the course of anti-MP treatment remains unclear.

**Methods:**

We retrospectively reviewed the medical records of 94 MPP children. Patients were classified into two groups. The long-term group (Group LT) was defined as bronchoalveolar lavage fluid (BALF) remained MP-positive by PCR after 30 days of the disease course. The non-long-term group (Group NLT) was defined as BALF became MP-negative by PCR within 30 days of disease and patients who only needed one bronchoscopy lavage therapy. MP loads, clinical outcomes were analyzed along with other clinical measurements.

**Results:**

The average levels of inflammatory markers such as C reactive protein and lactate dehydrogenase in Group LT were significantly higher than those in Group NLT. Airway and lung damage in Group LT were more severe than Group NLT. 28 patients developed necrotizing pneumonia and 8 patients developed pulmonary embolism in Group LT. Mean maximum MP loads in BALF were 10^7.46 ± 0.93^ and 10^4.86 ± 0.93^ in Groups LT and NLT, respectively. There was persistent MP DNA in Group LT, even lasted for 120 days. One severe MPP patient in Group LT had MP-associated bloodstream infection. After 3 months of follow-up, chest imaging revealed incomplete absorption of pulmonary consolidation in 33 patients of Group LT [including 13 airway obliterans (AO) patients] and in 7 patients of Group NLT (including 2 AO patients).

**Conclusion:**

MP loads of BALF were associated with the subsequent duration of MP DNA in lower airway. High MP loads and persistent long-term MP DNA in lower airway were associated with severity of pediatric MPP.

## Background

An increased number of refractory, severe, and even fatal *Mycoplasma pneumoniae* (MP) pneumonia (MPP) cases have been reported in recent year s[1–3]. Although the patient’s body temperature can return to normal after treatment, airway and pulmonary lesions are still severe in some refractory MPP (RMPP) patients. Furthermore, some RMPP patients have sequelae, mainly atelectasis, due to airway obliterans (AO )[4].

Cell-mediated immunity and cytokine responses against the pathogens, MP resistance to macrolides and airway hypersecretion have been reported in MPP [5, 6]. However some patients received timely treatment with sensitive antibiotics such as moxifloxacin, corticosteroid, and bronchoscopy lavage therapy (BLT), AO was still unavoidable. We observed airway hypersecretion and subsequent airway damage was severe in some children with RMPP, who required several treatments with BLT. Therefore, many factors of RMPP remain unclear, such as why airway damage persists, how long it takes to clear MP, and the course of anti-MP treatment. Elucidating these issues is important to help guide treatment strategies and further understand the underlying mechanisms of pediatric RMPP.

Here, we retrospectively analyzed MP loads in 94 MPP patients, including 90 RMPP patients. Fluorescence quantitative PCR (FQ-PCR) for MP gene detection was applied in all patients. Our studies suggested that MP could persist in the lower airway for up to 4 months, which is consistent with the severity of airway and pulmonary damage. In addition, MP culture was performed in 15 bronchoalveolar lavage fluid (BALF, MP loads > 10^5^ copies/mL) samples after 30 days of the disease course; 12 of 15 samples were positive, suggesting active MP infection. Moreover, MP was detected by FQ-PCR in the peripheral blood of one patient with severe MPP, which suggested a rare case of MP-associated bloodstream infection. To our knowledge, this is the first report of persistent MP infection and difficult MP clearance in the lower airway of human.

## Methods

### Study population

We retrospectively reviewed the medical records of 94 previously healthy MPP patients who were admitted in the Department of Respiratory Medicine II at Beijing Children’s Hospital affiliated to Capital Medical University, National Center for Children’s Health, between January and December 2017. The study was approved by the Ethics Committee of Beijing Children’s Hospital (No. 2017–23), and informed written consent was obtained from guardian of all the patients at the beginning of admission to our department. Patients with respiratory virus including adenovirus infection were excluded from current study.

In general, RMPP patients received treatment with azithromycin for 3–4 courses (nearly 1 month). In addition, it has been reported persistent MP infection of NHBE might last for up to 4 week s[7]. Therefore, patients were classified into two groups in this study. The long-term group (Group LT) was defined as BALF remained MP-positive by FQ-PCR after 30 days of the disease course. The non-long-term group (Group NLT) was defined as BALF became MP-negative by PCR within 30 days of disease and patients who only needed one BLT assessed by two clinicians.

The clinical and demographic data were collected and recorded for each patient. Variables included age, gender, disease course before first admission to our department, and inflammatory markers [the maximal white blood cells (WBC), C reactive protein (CRP, normal range < 8 mg/L), lactate dehydrogenase (LDH), normal range < 240 IU/L] within 15 days of disease course. Additionally the MP loads, findings of bronchoscopy and chest imaging, and treatment with anti-MP antibiotics and corticosteroid were recorded.

### Definitions

RMPP was diagnosed according to the following criteria: (1) patients with MPP had persistent fever and deterioration of clinical and radiological findings after 7 days of disease course; (2) patients were treated with macrolides for 7 days or more [6, 8].

**Fig. 1:**
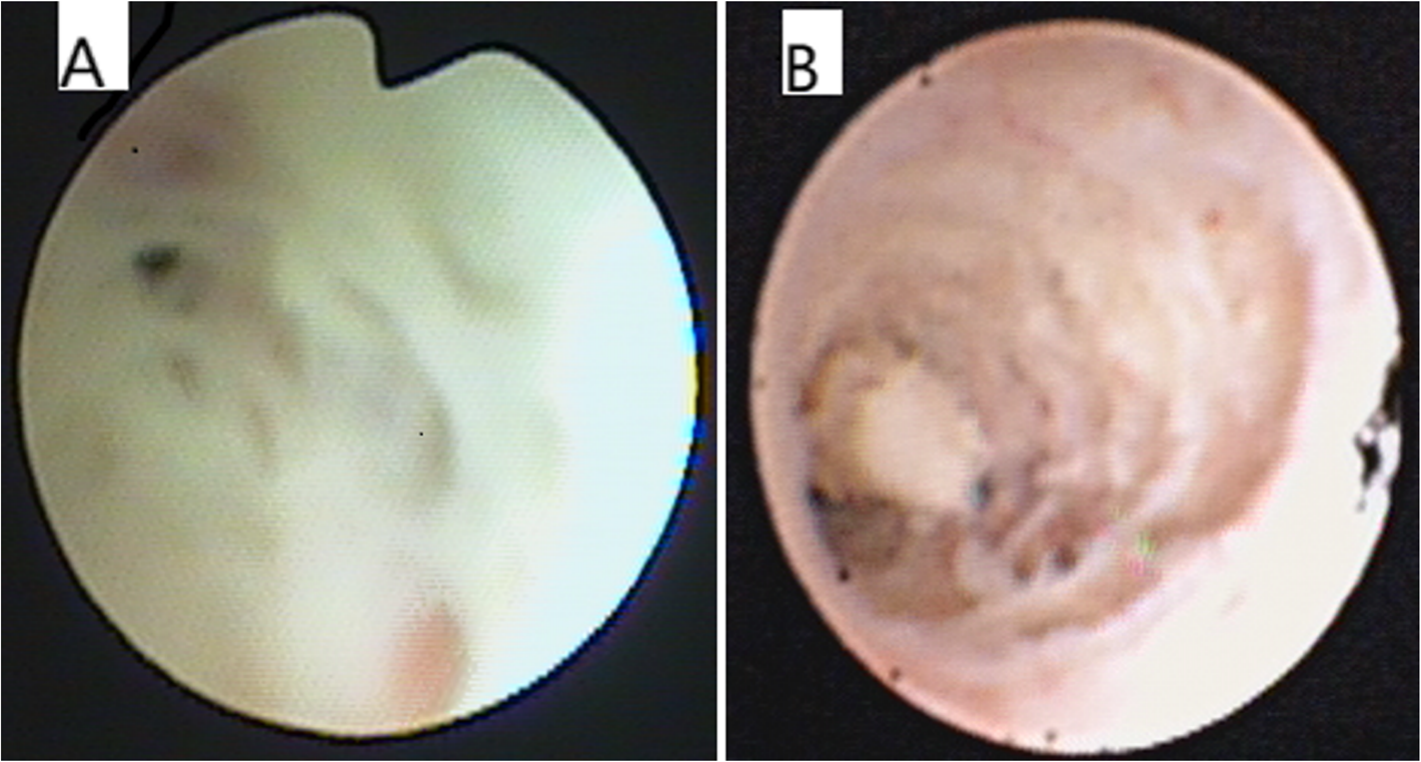
**a**: Fiberoptic bronchoscopy revealed AO (the obliteration of the lumen of bronchial branches, bronchial segments, or bronchial subsegments) in late stage of disease in RMPP patients. The rate of AO was 26% (13/50) and 6.8% (3/44) in Groups LT and NLT, respectively. **b**: Fiberoptic bronchoscopy revealed mucous plug at the early stage of disease in RMPP patients. The rate of mucus plug was 82% (41/50) and 6.8% (3/44) in Groups LT and NLT, respectively

The diagnosis of AO was based on the obliteration of the lumen of bronchi, bronchial branches, bronchial segments, or bronchial subsegments under bronchoscopy (Fig. 1a )[6]. The imaging findings of AO were atelectasis.

### Fiberoptic bronchoscopy and BALF collection

Fiberoptic bronchoscopy and BALF Collection were performed as previously reported [6].

### FQ PCR

FQ PCR was performed as previously reported [9]. The standard curves for each MP Quantitative was determined by analyzing serial 10-fold dilutions of stand red MP DNA (M129 strain, 1 copy/μl to 10^8^copies/μl). Each DNA of specimens and stand red was assayed in triplicate.

### MP genotyping and antimicrobial susceptibility testing

MP genotyping was performed by real-time PCR assay [10]. Minimum inhibitory concentrations (MICs) against 4 antibiotics (erythromycin, azithromycin, levofloxacin, tetracycline) were determined using SP4 broth (Remel) based on the micro-dilution methods (CSLI M43-A).

### MP culture of BALF samples

BALF was cultured in *Mycoplasma* selective liquid media (OXOID) at 37 °C. When the color of the media changed from red to yellow, approximately 0.1 ml of the suspension was transferred onto agar to subculture and purify the bacteria using the filtration-cloning technique for MP clinical isolates.

### Statistical analyses

The statistical analysis was performed in the SPSS version 17.0 (SPSS Inc., Chicago, Illinois, USA). All value data presented were expressed as mean ± standard deviation (SD), and student’s t-test was utilized for the comparison of these data between Group LT and Group NLT. The chi-square test was employed to compare categorical data. All statistical hypothesis tests were 2-sided, and *P* values < 0.05 were considered statistically significant.

## Results

### Study population

A total of 94 MPP patients (age range: 3 years 4 months–13 years 7 months) were enrolled in this study. The cohort was 50% male (*n* = 47). All patients in Group LT and 40 patients in Group NLT were finally diagnosed with RMPP. There was no significant difference in gender and age between Groups LT and NLT (*P* > 0.05) (Table 1).

**Table 1:** Demographic and clinical features, inflammatory markers, and the findings of bronchoscopy and chest imaging in all patients

	Group LT(*n* = 50)	Group NLT(*n* = 44)	*P* Value
Male/female	22/28	25/19	*P* = 0.215
Age (years)	7.23 ± 2.48	7.75 ± 2.28	*P* = 0.409
Disease course before first admission (days)	17.40 ± 13.33	9.27 ± 3.79	P < 0.001
Respiratory failure (n/%)	12/(24%)	0/(0%)	*P* = 0.001
WBC (×10^9^/L)	11.85 ± 4.61	8.47 ± 2.48	*P* < 0.001
CRP (mg/L)	104.96 ± 54.20	30.32 ± 20.64	*P* < 0.001
LDH (IU/L)	681.00 ± 298.24	330.11 ± 81.37	*P* < 0.001
Bronchoscopy findings			
Early stage			
Mucous plug (n/%)	41/(82%)	3/(6.8%)	*P* < 0.001
Mucus necrosis (n/%)	29/(58%)	3/(6.8%)	*P* < 0.001
Late stage			
Stenosis (n/%)	13/(26%)	3/(6.8%)	*P* = 0.014
AO (n/%)	13/(26%)	2/(4.5%)	*P* = 0.005
Chest imaging findings			
Consolidation (>one pulmonary lobe) (n/%)	46/(92%)	0/(0%)	*P* < 0.001
Pleural effusion (n/%)	34/(68%)	2/(4.5%)	P < 0.001
Pulmonary embolism (n/%)	8/(16%)	0/(0%)	*P* < 0.001
NP (n/%)	28/(56%)	1/(2.3%)	*P* < 0.001

Short names: *WBC* white blood cells, *CRP* C reactive protien, *LDH* lactate dehydrogenase, *AO* Airway obliterans, *NP* necrotizing pneumonia

### Clinical characteristics

The average duration of disease before hospitalization was 17.40 ± 13.33 days and 9.27 ± 3.79 days in Groups LT and NLT, respectively (*P* < 0.001).

In Group LT, most patients resided from all over the country and 12 patients had type I respiratory failure, which suggested patients in this group were severe and difficult to treat. Mean WBC count, CRP level, and LDH concentration were 11.85 ± 4.61 × 10^9^/L, 104.96 ± 54.20 mg/L, and 681.00 ± 298.24 IU/L, respectively.

In Group NLT, mean WBC count, CRP level, and LDH concentration were 8.47 ± 2.48 × 10^9^/L, 30.32 ± 20.64 mg/L, and 330.11 ± 81.37 IU/L, respectively.

A clear statistical difference was observed in inflammatory markers (WBC, CRP, LDH) between the two groups (*P* < 0.001) (Table 1).

### Bronchoscopy findings

**Fig. 2:**
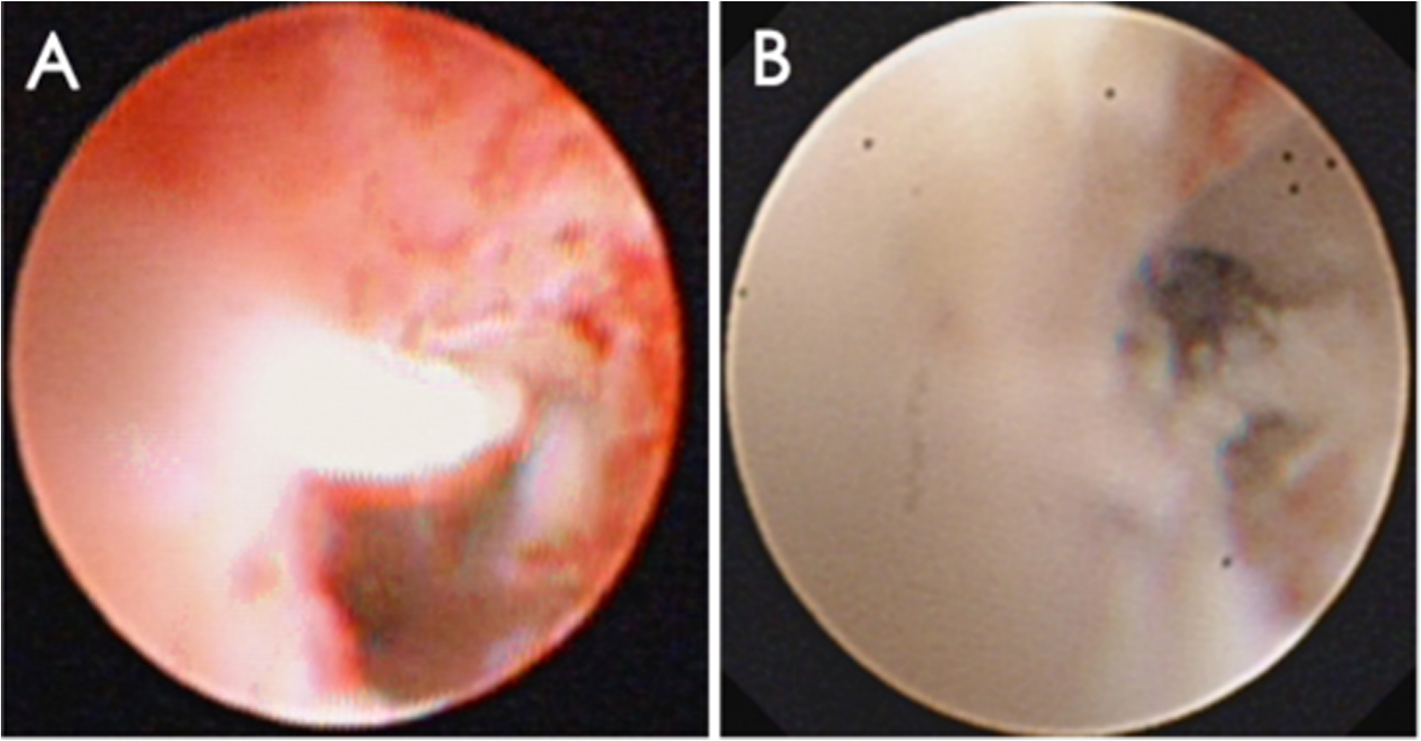
**a**, **b**: Fiberoptic bronchoscopy revealed airway mucous necrosis at the early stage of disease in RMPP patients. The rate of mucus necrosis was 58% (29/50) and 6.8% (3/44) in Groups LT and NLT, respectively

In Group LT, bronchoscopy carried out at the early stage of disease revealed mucus plug (Fig. 1b) and airway mucous necrosis (Fig.2) in 41 (including plastic bronchitis in 6 patients) and 29 patients, respectively. In the late stage of disease, 13 patients had stenosis and 13 patients had AO.

In Group NLT, three patients had a mucus plug and three patients had mucous necrosis in the early stage of disease. In the late stage of disease, bronchoscopy revealed stenosis and AO in three and two patients, respectively.

A clear statistical difference was observed in airway damage between the two groups (*P* < 0.05) (Table 1).

### Chest imaging findings

**Fig. 3:**
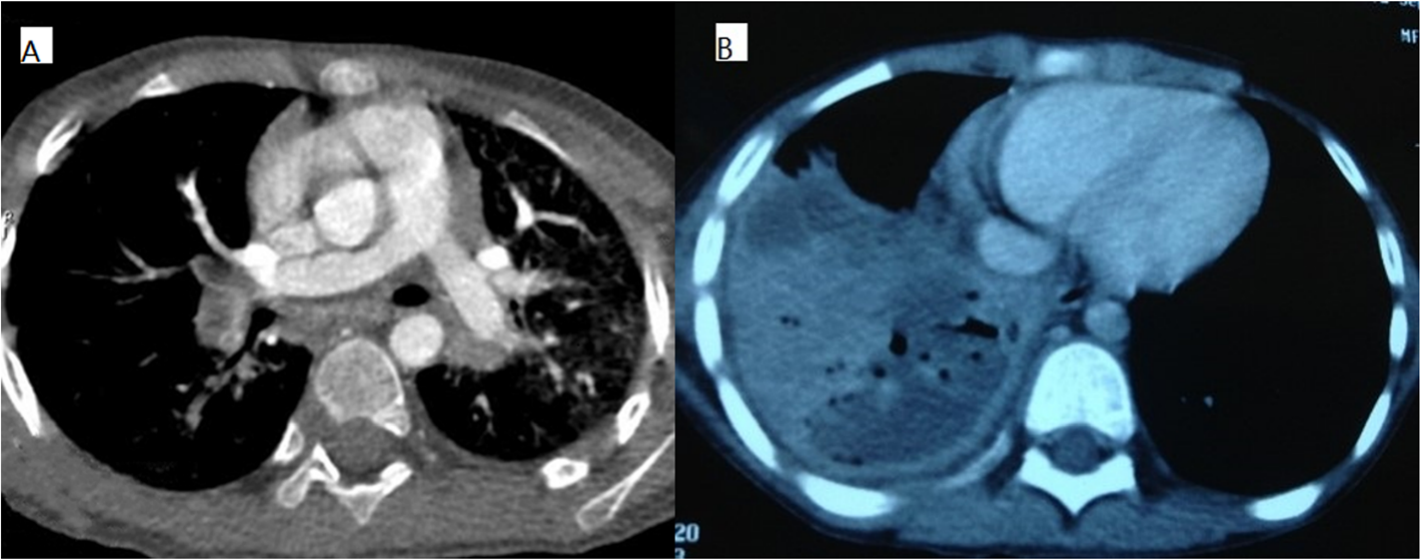
**a**: Enhanced pulmonary CT revealed bilateral pulmonary embolism (PE) in 8 patients (16%, 8/50) of Group LT between 11 and 29 days of disease. No one (0%) had PE in Group NLT. **b**: Chest CT revealed low density lesions and multiple cavities within high density consolidation in 28 patients (56%, 28/50) of Group LT between 14 and 60 days of disease. Only one patient (2.3%,1/44) had NP in Group NLT

In Group LT, chest imaging at the early stage of disease revealed consolidation with high density (2/3–1 pulmonary lobe in 4 patients; ≥1 lobe in 46 patients) in 50 patients and pleural effusion in 34 patients. Additionally, chest imaging revealed pulmonary embolism in 8 patients between 11 and 29 days of disease and necrotizing pneumonia (NP) in 28 patients between 14 and 60 days of disease (Fig. 3).

In Group NLT, chest imaging revealed consolidation with high density (< 1/2 pulmonary lobe in 4 patients, 1/2–2/3 lobe in 24 patients, and 2/3–1 lobe in 11 patients) in 39 patients and bronchiolitis in 5 patients. Only two patients also had a small amount of pleural effusion.

A clear statistical difference was observed in lung damage between the two groups (*P* < 0.05) (Table 1).

### MP loads by PCR

#### MP loads in paired throat swab and BALF collected at the same time point

In Group LT, throat swabs were performed in 20 patients 22–45 days after disease; 15 patients were MP-positive (including one patient with 10^5^ copies/mL in 45 days) and 5 patients were MP-negative. Additionally, MP loads in throat swabs were lower than those in BALF. All BALF samples were MP-positive.

In Group NLT, throat swabs were performed in 10 RMPP patients 18–22 days after disease; 3 patients were MP-positive and 7 patients were MP-negative. Five BALF samples were MP-positive.

These results suggest that patients in Group LT had long-term persistent upper airway infection and MP was cleared from the upper airway before the lower airway (Table 2).

**Table 2:** Bacterial MP loads and duration of MP infection

	Group LT (n = 50)	Group NLT (n = 44)
Positive MP loads in throat swab (n/disease course/ copies/ml)	15/(22–45)days/10^3–5^	3/(18–22)days/10^2–3^
Negative MP loads in throat swab (n/disease course)	5/(26–31)days	7/(19–22)days
Highest MP loads in BALF		
High load (n/%/copies/ml)	44/(88%)/ 10^7–8^	2/(4.54%)/10^7–8^
Middle load (n/%/copies/ml)	4/(8%)/10^6^	5/(11.36%)/10^6^
Low load (n/%/copies/ml)	2/(4%)/10^5^	37/(84.09%)/10^2–5^
Highest MP loads in BALF (copies/mL)	10^7.46 ± 0.93^	10^4.86 ± 0.93^
Duration of MP infection in BALF		
Within 31–40 days (n/copies/ml)	17 /(10^2–7^)	–
Within 41–50 days (n/copies/ml)	19/(10^2–7^)	–
Within 51–60 days (n/copies/ml)	9/(10^2–7^)	–
Within 61–90 days (n/copies/ml)	4/(10^2–4^)	–
Cases 1 and 2	NP	–
Cases 3 and 4	AO	–
120 days (n/copies/ml)	1/(10^3^)	–
Case 5	NP	–
Duration of negative MP in BALF (n/disease course)	–	17/(15–30)days

Short names: *MP Mycoplasma pneumoniae, BALF* bronchoalveolar lavage fluid, *AO* airway obliterans, *NP* necrotizing pneumonia

#### Maximum MP loads in BALF

MP loads in BALF were classified into three groups as follows: high bacterial loads (≥10^7^ copies/mL), moderate bacterial loads (10^6^ copies/mL), and low bacterial loads (< 10^6^ copies/mL).

In Group LT, high, moderate, and low MP loads were found in 44 (88%, 44/50), 4 (8%, 4/50), and 2 (4%, 2/50) patients, respectively. In Group NLT, high, moderate, and low MP loads were found in 2 (5%, 2/44), 5 (11%, 5/44), and 37 (84%, 37/44) patients, respectively. Mean MP loads in BALF were 10^7.46 ± 0.93^ and 10^4.86 ± 0.93^ in Groups LT and NLT, respectively (*P* < 0.001), which suggest MP loads in BALF are associated with the duration of MP infection.

#### Duration of MP infection in BALF

In Group LT, MP DNA gradually declined over time in most patients. However, in some patients, MP copy numbers initially increased and then declined. MP was detected by PCR within 31–40 days, 41–50 days, 51–60 days, and 61–90 days after disease onset in 17, 19, 9, and 4 patients, respectively. Cases 1–4 (2 NP patients and 2 AO patients) had detectable MP loads 61–90 days after disease onset. Moreover, 120 days after disease onset, MP was detected by PCR in an NP patient (Case 5, 5.98 × 10^3^ copies/mL).

In Group NLT, 17 patients received more than two treatments of BLT and became MP-negative by PCR within 30 days of the disease. The remaining 27 patients received BLT once and were MP-positive by PCR.

These results demonstrate persistent long-term infection in the lower airway of patients in Group LT.

#### MP loads in peripheral blood

MP PCR was performed in 20 RMPP patients in Group LT within 7 days of disease. MP was detected in only one patient with severe RMPP (Case 6, 3.34 × 10^3^ copies/mL), which suggested a rare MP-associated bloodstream infection. This patient had type I respiratory failure and a pulmonary embolism.

### Detection of MP genotyping, antimicrobial susceptibility testing and MP culture in BALF

MP genotyping, antimicrobial susceptibility testing, and MP culture was performed in 30 patients of Group LT and 11 patients of Group NLT. Genotype 2 was detected in 3 patients of Group LT and 1 patient of Group NLT. Genotype 1 was detected in the other 37 patients. Only one strain (Group NLT) was susceptible to the macrolide (genotype 2). The other 40 strains were macrolide-resistant and carried the A2063G mutation. No strains with resistance to the levofloxacin and tetracycline were identified. Moreover, MP culture was performed in 15 patients of Group LT whose MP loads were > 10^5^ copies/mL within 31–60 days after disease onset. Twelve of 15 patients were found to be MP-positive.

### Treatment and clinical outcomes

All patients were treated with macrolides and BLT and followed up for at least 3 months. Four non-RMPP patients in Group NLT were not treated by glucocorticoid.

In Group LT, moxifloxacin and doxycycline were also added to the treatment course in 14 patients and 2 patients, respectively. Azithromycin, which has anti-inflammatory effects, was administered for 3–6 months in 23 patients. Twenty-one patients whose CRP levels were > 100 mg/L received high-dose methylprednisolone therapy (10–30 mg/kg/d for 3 days); after 3 months, chest imaging findings were normal in only 7 patients. These seven patients had started corticosteroid treatment 7–9 days into the disease course. In Group LT, chest imaging after 3 months of treatment revealed incomplete absorption of pulmonary lesions in 33 patients, including 13 AO patients.

In Group NLT, chest imaging after 3 months of treatment revealed incomplete absorption of pulmonary consolidation in seven patients, including two AO patients.

No obvious side effects were observed in any patients. Furthermore, there were no deaths in our study.

Taken together, these results indicate MP infection was difficult to clear, and absorption of lung lesions was very slow, especially in Group LT.

## Discussion

Pediatric MPP is a significant public health problem. The clinical severity of MP infection is associated with MP loads in oropharyngeal secretion s[11, 12]. Nilsson et al. observed that the majority of patients with MP-positive throat swabs by PCR had persistent, sometimes long-term, upper airway infections (median time of carriage of MP DNA was 7 weeks after disease onset )[13]. However, to our knowledge, there is no report on the duration of MP infection in the lower airway of human. BALF is the most reliable specimen for diagnosing and assessing lower respiratory tract infection. In our study, all patients received BLT and some patients received BLT several times. We found MP was cleared in the upper airway earlier than the lower airway, and MP loads in BALF in the early stage of disease were associated with the subsequent duration of MP infection in the lower airway. Additionally, inflammatory markers (WBC, CRP, and LDH), airway and lung injury, and pulmonary complications were highly associated with MP loads and persistent MP DNA in BALF. Strikingly, in Group LT, all patients had persistent MP DNA even for up to 120 days, and some patients still had high MP loads 50 days after disease onset. Given that PCR cannot distinguish live bacteria from dead bacteria, we also performed MP culture for 15 patients whose MP loads were > 10^5^ copies/mL; 12 of these patients were MP-positive, which suggested persistent long-term MP infection in the lower respiratory tract.

In our study, high MP loads and prolonged infection were mainly accompanied by airway damage, such as epithelial desquamation, necrosis, and AO, and pulmonary damage, such as consolidation and necrosis (sometimes including recurrent low or moderate fever in few patients). These findings suggested persistent MP activation and chronic respiratory infection rather than a state of chronic MP colonization. A previous study reported that adequate antibiotic treatment does not shorten the period of persistence,[13] which was confirmed in the present study.

It has been reported that MP clearance was difficult in animal experiments or in vitro*.* Airway hypersecretion, epithelial desquamation, and remodeling cause AO, which compromises further clearance in vitro*,*[7] which was consistent with our present study. Other studies have shown that MP is resistant to killing by neutrophils, suggesting that the bacterium might circumvent bactericidal activity of neutrophil extracellular trap s[14]. B cells and MP-specific antibodies are also crucial for its clearance in the lungs of mic e[15]. Interleukin (IL)-1 plays an important role in recruiting and activating lung innate immune cells that are critical for MP clearance [16]. MP respiratory tract infection in IL-12 knockout mice results in improved bacterial clearance and reduced pulmonary inflammation such as neutrophilic alveolar infiltrate and airway obstructio n[17]. Intranasal IL-12 therapy inhibits MP clearance and sustains airway obstruction in murine pneumoni a[18]. In addition, host genetic background was also found to be important for MP disease severit y[19, 20]. However, the underlying mechanisms of persistence remain unclear.

Macrolide-resistant (MR)-MP strains are emerging and becoming increasingly common, especially in Eastern Asia, and have been reported at a rate of > 90% in Beijing from 2008 to 201 2[21]. In our present study, type 1 was the main genotype and the total MR-MP rate was high up to 97.6% (40/41). In addition, the MR-MP rate of genotype 1 and genotype 2 was 100% (37/37) and 75% (3/4) respectively, which was consistent with our previous report [22]. Macrolides are still used as first-line agents at high frequencies in countries with a high burden of MR-MP strains, such as China and Japan, mainly because of their low minimum inhibitory concentrations against the bacteria, anti-inflammatory effect and low toxicity in young children. Yang et al. reported that timely azithromycin treatment may be ineffective in treating MR-MP pneumonia in vivo, but we are unable to withhold antibiotic treatment to confirm this hypothesis due to ethical issues. However, the early use of azithromycin aids in reducing extrapulmonary complications in MPP [23]. Yoon et al. found macrolide treatment for macrolide-susceptible MPP did not contribute to significant clinical improvement compared to no antimicrobial treatment [24]. Kutty et al. reported that among MP PCR-positive children, length of stay was similar between children who did and did not receive an antibiotic with MP activity [25].

To date, no tetracycline or fluoroquinolone resistance has been reported in MP clinical isolates. Although some of our patients received timely treatment with moxifloxacin or doxycycline even for several courses, MP remained difficult to clear. Yang et al. reported anti-MP-antibiotics might have limited effects on MPP,[26] which was consistent with our study.

A previous study showed that 3-day methylprednisolone pulse therapy which was initiated 7–15 days after disease onset could be applied for RMPP treatment despite appropriate antibiotic therapy and appeared to be efficacious in 12 children [27]. However methylprednisolone pulse therapy was given 9 days after disease onset in our 14 patients in Group LT, chest imaging findings remained abnormal after 3 months of treatment. Therefore, the underlying pathogenesis of RMPP is still unclear.

Compared with C57BL/6 mice, BALB/c mice exhibited host-dependent MP infection-related airway obstruction associated with chemokine, [20] which suggested some pediatric RMPP patients were highly reactive to MP. Community-acquired respiratory distress syndrome toxin induces exfoliation of mucosal cells and ciliostasis [28]. Airway epithelial remodeling during persistent MP infection may involve epithelial-to-mesenchymal transition in a human airway epithelium model [7]. Therefore, high MP loads and epithelial desquamation, subsequent persistent MP infection, epithelial remodeling, and AO can lead to a vicious cycle of MP disease.

We found that MP may survive for a long time, especially in RMPP patients who suffer from MP associated NP, AO, or pulmonary embolism. Furthermore, although MP-associated bloodstream infection is rare, it can occur.

Our study has several limitations. The numbers of blood samples and throat swabs were small. The reason for the small number of blood samples is that the MP-negative rate is very high, in RMPP, thus clinicians do not often request blood samples for MP detection. The number of children treated with moxifloxacin and doxycycline is small. Additionally, in some RMPP patients of Group LT, we did not store the last collected BALF sample. Therefore, the actual duration of persistence is likely underestimated.

## Conclusions

Our study confirms persistent long-term MP DNA and difficult MP clearance in the lower airway of RMPP patients. Additionally, patients with MP loads ≥10^7^ copies/mL in BALF are perhaps at high risk of RMPP and may be more likely to develop sequelae. Lastly, patients who have high levels of inflammatory markers, such as CRP and LDH, and high-density consolidation (especially more than one pulmonary lobe) on imaging may also have higher MP loads and subsequent persistent MP airway infection that is accompanied by NP, AO, or pulmonary embolism.

## Data Availability

The datasets generated during and/or analysed during the current study are available from the corresponding author on reasonable request.

## Abbreviations

AO: Airway obliterans
BALF: Bronchoalveolar lavage fluid
BLT: Bronchoscopy lavage therapy
CRP: C reactive protein
LDH: Lactate dehydrogenase
LT: Long-term
MP: *Mycoplasma pneumoniae*
MPP: *Mycoplasma pneumoniae* pneumonia
MR: Macrolide-resistant
NLT: Non-long-term
RMPP: Refractory MPP
WBC: White blood cells
